# The Bidirectional Interactions between Resveratrol and Gut Microbiota: An Insight into Oxidative Stress and Inflammatory Bowel Disease Therapy

**DOI:** 10.1155/2019/5403761

**Published:** 2019-04-24

**Authors:** Yaolian Hu, Daiwen Chen, Ping Zheng, Jie Yu, Jun He, Xiangbing Mao, Bing Yu

**Affiliations:** ^1^Key Laboratory of Animal Disease-Resistant Nutrition, Sichuan Province, China; ^2^Key Laboratory of Animal Disease-Resistant Nutrition, Ministry of Education, China; ^3^Animal Nutrition Institute, Sichuan Agricultural University, Ya'an, 625014, China

## Abstract

Dysbiosis and oxidative stress in the gut have contributed to the progression of intestinal inflammatory bowel disease (IBD). The current study has reported that enteric bacteria mediate redox homeostasis through the regulation of reactive oxygen species (ROS) production. Resveratrol, one of the most abundant polyphenols, with poor oral bioavailability, is considered as a scavenger of ROS and other free radicals. Recent studies have shown that resveratrol effectively enhances the growth of* Lactococcus lactis* and inhibits the growth of* Enterococcus faecalis*. (1) In terms of the two-way relationship between gut microbiota and resveratrol, resveratrol modulates gut microbiota; (2) in terms of resveratrol biotransformation by gut microbiota, we speculate that gut microbiota could be a target of resveratrol to maintain gut homeostasis. Here, we reviewed the current researches about the cellular signaling pathways in intestinal epithelial cells triggered by gut microbiota in response to oxidative stress. These results suggest that the modulation of the gut microbiota through resveratrol supplementation appears as a promising potential approach for the therapy of inflammatory bowel disease.

## 1. Introduction

Oxidative stress is caused by an imbalance between reactive oxygen species (ROS) production and cellular antioxidant capacity with accumulation of excessive ROS in cells [1, 2]. Prolonged oxidative stress plays a key role in the initiation and development of inflammatory bowel disease (IBD), including Crohn's disease (CD) and ulcerative colitis (UC) [3–5]. IBD is an inappropriate immune response caused by a range of genetic, microbial, and environmental factors, characterized by chronic inflammation with alternating periods of remissions and relapses [6–8]. Recent evidence further identified that oxidative stress also appears to play a pathogenic role in chronic inflammatory diseases [9–11]. The inhibition of NF-E2 related factor-2 (Nrf2) attenuated anti‐oxidative stress pathway and induced inflammation in Nrf2 knockout mice [12]. The intestinal mucosae of patients with IBD have high levels of ROS and decreased antioxidant defense capacity [13]. Excessive production of ROS can increase membrane permeability and cellular stress, finally resulting in the expansion of facultative anaerobic bacteria, gut barrier dysfunction, and inflammation [14, 15]. Moreover, experimental UC in mice is attenuated by antioxidant interventions [16]. Notably, certain intestinal epithelial cells have been reported to rapidly generate ROS in response to microbial signals [17]. The enteric bacteria in the intestinal lumen have been identified as a large source of redox-based effector signals. They regulate multiple cell signaling via promoting the generation of ROS in intestinal epithelial cells, leading to the initiation and progression of many diseases such as IBD, metabolic syndrome, and cancer [18, 19]. The pathogenic bacteria-induced ROS will cause DNA damage in epithelial cells, which can give rise to genomic instability and the dysregulation of epithelial barrier function [20, 21]. It is generally accepted that chronic inflammation is associated with the inability of the gut immune system to manage microflora and the alteration in the microbial composition in the gastrointestinal tract [22–25]. Furthermore, the metabolism disorder of bacterial fermentation products short-chain fatty acids (SCFAs) may stimulate the immune responses, resulting in increased intestinal epithelial permeability [26]. In all, the ROS overproduction in intestinal epithelial cells is the main consequence of intestinal dysbiosis that can be considered a significant risk factor of IBD. Thus, targeting gut microbiota to improve of oxidative stress and inflammation in the gut could be a promising potential therapy for IBD.

Resveratrol (3,5,4′-trihydroxy-trans-stilbene), a nonflavonoid polyphenol compound, is found in various plants, including* Vitis vinifera*,* Arachis hypogaea*, and* Polygonum cuspidatum* [27, 28]. It is proved to be a potent antioxidant, antibacterial, antiobesity, anti-inflammatory, and anticancer agent in* in vitro* and* in vivo* experiments [29–33]. Notably, resveratrol is considered as a strong scavenger of ROS and other free radicals [34]. In a review, Manach et al. [35] proposed that polyphenols are the major dietary antioxidants present in the colon, as other antioxidants, such as vitamins E and C, are absorbed in the small intestine. Although resveratrol has shown beneficial effects on host metabolism, measurements of plasma levels of the parent drug suggest that resveratrol exhibits poor bioavailability and high rate of metabolism when administered orally [36, 37]. Resveratrol can be metabolized by hepatic and gut microbial enzymes [38, 39]. The metabolite of resveratrol, dihydroresveratrol (DH-RES), is formed in cecum, colon, and rectum by gut microbiota fermentation, and the level of DH-RES is much higher than that of resveratrol. Therefore, this metabolite may also contribute to pharmacological activity in the human large intestine [36]. Besides, many studies have assessed the effect of resveratrol on gut microbiota diversity and composition, including inhibiting the growth of* Enterococcus faecalis*, increasing the Bacteroidetes-to-Firmicutes ratios, and increasing the* Lactobacillus* and* Bifidobacterium* populations [40, 41]. Here, we discuss the cellular signaling triggered by enteric bacteria in host defense and disease development. In addition, we review the reciprocal interactions between resveratrol and gut microbiota, as well as the new evidence for the treatment of oxidative stress and inflammatory bowel disease.

## 2. The Role of Gut Microbiota in IBD

The intestine contains a complex microbial community of 100 trillion bacterial cells with more than 1,000 species, most of which are beneficial to our health [42, 43]. The gut microbiota affect human metabolism due to their ability to interact with receptors on gut epithelial cells and other effector cells [25, 44, 45]. Given the development of novel diagnostic and therapeutic approaches for human diseases, a critical need exists for a deeper understanding of the underlying mechanism of cellular signaling induced by gut microbiota, and the symbiotic relationship between gut microbiota and the host.

**Figure 1:**
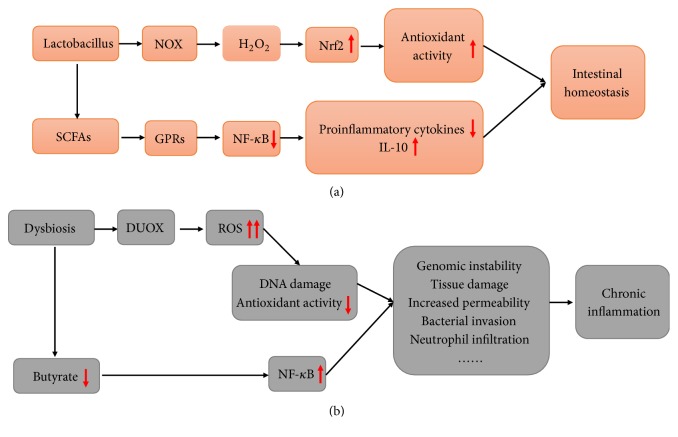
The associations between gut microbiota, intestinal inflammation, and oxidative stress. (a) Intestinal homeostasis is associated with enteric bacteria residing in the intestinal lumen. (b) Dysbiosis and oxidative stress in the gut have been shown as contributors of the pathogenesis of intestinal diseases.

In response to symbiotic microbes, intestinal epithelial cells can use NADPH oxidase (NOX) family to generate ROS, which have been detected in different multicellular organisms [46, 47]. NOX-produced H_2_O_2_, a major nonradical ROS production in the epithelial cells after the formylated peptide receptors sense and bind to a specific N-formyl group of the* Lactobacilli*, is well documented as a second messenger in signal transduction networks. These changes in H_2_O_2_ do not cause a significant imbalance between oxidant production and antioxidant levels [48–50]. H_2_O_2_ generated by the cells can oxidize redox-sensitive cysteine residues on Kelch-like ECH-associated protein 1 (Keap1), resulting in the activation of Nrf2 (see Figure 1) [51]. Of note, high levels of H_2_O_2_ further oxidize thiolate anions of the peroxidatic Cys to sulfinic (SO_2_H) or sulfonic (SO_3_H) species. This irreversible modification can lead to cellular damage and oxidative stress [52]. In fact, the diverse biological outcomes of different ROS depend on the specificity and selectivity of ROS on their targets and the compartmentalization of ROS production in cells [53].* Lactococcus lactis* was found to diminish oxidative stress, by releasing cytoplasmic superoxide dismutase A (SodA) due to host lysozyme-mediated lysis at inflamed colonic sites [54]. Interestingly,* Lactobacillus* appears to modify the natural course of the disease through the upregulation of Nrf2-dependent antioxidant enzymes.* Enterococcus faecalis* near the oxygenated colonic luminal surface generates extracellular O^2−^ at high rate causing intestinal injury [23, 55]. For example, germ-free interleukin-10 knockout (IL-10 KO) mice developed IBD after colonization with* Enterococcus faecalis* but not in the* Lactococcus lactis *colonized mice [56]. Indeed, unlike* Lactococcus lactis*, intestinal ROS overproduction is induced by opportunistic pathogens under certain conditions, and this can aggravate intestinal damage. The bacterial-derived uracil is responsible for dual oxidase (DUOX)-dependent ROS generation in human mucosal epithelial cells, which can lead to overexpression of DUOX2 and oxidative stress in the gut (see Figure 1) [57]. Oxygen radical generation by opportunistic pathogens promotes epithelial cell DNA damage and exacerbates intestine inflammation [58]. Taken together, the microbiota composition is thought to be a major determinant of health and disease of the host, because of the potential of intestinal microbiota to modulate ROS production and antioxidant defense.

Culture-independent methods to analyze the gut microbial composition allow a more detailed understanding of the alterations in gut microbe and IBD. The high levels of mucosa-associated bacteria were detected in those patients [59, 60]. As mentioned above, the studies have demonstrated an association between microbiota composition and redox state of enterocytes; that is, several enteric bacteria could produce extracellular ROS, which may dysregulate intestinal homeostasis (see Figure 1). Moreover, anaerobic intestinal bacteria can downregulate proinflammatory cytokines expression and modulate the host mucosal immune response by inhibiting peroxisome proliferator-activated receptor gamma-mediated nuclear factor-kappaB (NF-*κ*B) activation [61, 62]. Furthermore, anaerobic bacterial fermentation gives rise to the production of SCFAs, including acetate, propionate, and butyrate. SCFAs have been suggested to bind to the intestinal epithelial cell-surface G-protein-coupled receptors (GPRs) in the surface of intestinal epithelial cells, such as GPR41, GPR43, and GPR109A [63–66]. They maintain intestinal homeostasis by suppressing NF-*κ*B activation, reducing the production of proinflammatory factors, increasing intestinal mucus synthesis, and decreasing the intestinal epithelial permeability (see Figure 1) [67–69]. Moreover, the metabolism of butyrate in the colonocytes has been shown to drive the oxygen consumption, which leads to a significant reduction of H_2_O_2_-induced DNA damage [26, 70]. A hypoxic environment induced by the oxygen consumption is also essential for preventing the expansion of facultative anaerobic bacteria. However, continued oxidative stress can alter the colonocyte metabolism and damage the hypoxic environment in large intestine, leading to the expansion of facultative anaerobic bacteria during dysbiosis [71]. The disorder of butyrate metabolism can give rise to an increasing number of macrophages and NF-*κ*B activation in colonocytes of patients with UC (see Figure 1) [72].

Several clinical studies have shown that the fecal microbiota transplantation (FMT) from the healthy donor promotes intestinal microbiota recovery and inflammation resolution in IBD patients [73, 74]. Therefore, a new potent approach by normalizing the intestinal dysbiosis and improving redox imbalance may open a new era for patients with chronic inflammation in the gut.

## 3. The Relationship between Resveratrol and Gut Microbiota

Resveratrol and its derivatives have shown therapeutic potential for the prevention and treatment of different chronic diseases such as diabetes and IBD [75–78]. Resveratrol, as a phytoalexin, shows antioxidant and anti-inflammatory activities to improve oxidative stress and chronic inflammation [30, 79]. For instance, resveratrol protects the porcine intestinal epithelial cell line (IPEC-J2) from mycotoxin-induced increases in intracellular ROS level and cell damage through the regulation of the Nrf2 signaling pathway [80]. The supplementation with 500 mg resveratrol can reduce levels of proinflammatory mediators and inhibit activity of NF-*κ*B in patients with active UC [81]. However, the low water solubility of resveratrol leads to poor oral bioavailability, which has limits for resveratrol's concentration in plasma [82, 83]. It seems to be a controversial issue about multiple biological activities of resveratrol. It can be speculated that enteric bacteria might represent a major contributor to the effects of resveratrol on the functional modifications of host cells [35, 84]. Using ultrahigh-performance liquid chromatography (UHPLC), coupled with a linear ion trap mass spectrometer, this hypothesis has been confirmed by these determinations of resveratrol metabolites in plasma and colon. Resveratrol was shown to be metabolized into various derivatives by pharmacokinetic profiling [85]. Resveratrol cannot be absorbed in the native form but is present at a very low concentration in plasma in conjugated forms such as sulfate and glucuronide conjugates [86–88]. An accumulation of resveratrol in the large intestine after oral administration has been reported, because of its poor absorption [37, 39]. It has been reported that resveratrol can modulate gut microbial composition while microbiota can also regulate resveratrol biotransformation [89, 90].

However, it is debated whether the effects of resveratrol on gut microbiome modulation are enough to be responsible for its antioxidant and anti-inflammatory activities and these effects are associated with the improvement of gut diseases in the process of bacteria-mediated ROS overproduction. The definitive mechanisms remain to be obtained. Based on the current evidence, the potential mechanisms will be discussed in the following sections.

### 3.1. Alterations of the Intestinal Microbiota by Resveratrol

Resveratrol is known to modulate the composition of the gut microbiota with a decrease in opportunistic pathogenic bacteria* in vivo*. Yang et al. [91] showed a significant decrease in the diversity of the gut microbiota and an increase in oxidative stress in high-fat-diet-fed rats compared with that in controls. Resveratrol supplementation (400 mg resveratrol per kg of feed for 8 weeks) increases the population of butyrate producer* Blautia* and* Dorea* in the* Lachnospiraceae* family. However, they noted that the supplementation of resveratrol had no significant influence on the levels of SCFAs. SCFAs are rapidly absorbed in the colon. This may result in the inaccuracy of SCFAs measurement. Additionally, resveratrol administration decreased the population of Bacteroidetes. Most* Bacteroides*, as antibiotic-resistant bacteria, may become highly pathogenic bacteria. The increased levels of* Bacteroides* can result in inflammation [92]. However, Qiao et al. [41] demonstrated that resveratrol supplementation (200 mg/kg/d for 12 weeks) promotes a higher Bacteroidetes-to-Firmicutes ratio in Western-diet-fed mice. Species (rats vs. mice) and/or housing environments (isolated vs. conventional) being studied could potentially explain the differences in specific microbial changes. In addition, the number of* Lactobacillus* and* Bifidobacterium* was significantly increased in resveratrol-fed animals. These genera, as noted above, are closely linked with redox signaling in mucosal epithelial cells, which plays a critical role in maintaining gut homeostasis. Moreover,* Enterococcus faecalis*, which is linked with the high levels of extracellular O^2−^, was significantly decreased in resveratrol-fed mice. Similarly, recent evidence suggests that the relative abundance of* Bacteroides*,* Lactobacillus*,* Bifidobacterium*, and* Akkermansia* is increased with resveratrol supplementation [93]. Previous studies have also shown that resveratrol administration leads to the increases in the number of* Lactobacilli *and the decrease in the number of* Enterococcus faecalis *and* Escherichia coli *species in high-fat-fed mice. Both* Escherichia coli *and* Enterococcus faecalis* are positively correlated with colonic ROS and MDA levels, while several bacteria such as* Lactobacilli *are significantly associated with colonic T-AOC [94]. Cellular studies also indicated an antimicrobial activity of natural phenolic compounds such as resveratrol and kaempferol, which is a growth inhibition of* Enterococcus faecalis* [95]. Thus, it is speculated that resveratrol alleviates oxidative stress by ROS and subsequent intestinal damage through gut microbiota (see Table 1).

A previous study, using the dextran sulfate sodium-induced colitis (DSS-colitis) rat model, has demonstrated that resveratrol treatment (1 mg/kg/day for 25 days) provides beneficial effects on the colon, including altering the expression of inflammation-associated genes, protecting the colonic mucosa architecture, and modulating intracellular signaling such as NF-*κ*B signaling pathway [84]. In addition, resveratrol administration can also restore normal intestinal microbiota in bacterial composition under DSS treatment. These include some anti-inflammatory gut microbiota, such as* Lactobacilli* and* Bifidobacteria. *More recently, Wellman et al. [96] reported that aged mice with deletion of Sirtuin1 in the intestinal epithelium (SIRT1 iKO mice) exhibited a reduced abundance of* Bacilli*, particularly* Lactobacillus*. Compared to wild-type DSS-colitis mice, aged SIRT1 iKO mice experienced enhanced rectal bleeding, increased colonic shortening, and elevated colonic crypt erosion after DSS challenge. Therefore, SIRT1 has been shown to improve intestinal barrier function and maintain epithelial cell homeostasis. Interestedly, depletion of gut microbiota by the antibiotic cocktail in SIRT1 iKO mice shows minimal colonic spontaneous inflammation. Hence, SIRT1 deficiency-induced intestinal mucosal damage is highly associated with alterations in gut microbiota upon DSS treatment. In view of the proved associations between gut microbiota, intestinal inflammation, and SIRT1, among them, gut microbiota is the main target of SIRT1 to reduce inflammation markers. Resveratrol, a well-known activator of SIRT1, has been shown to significantly increase SIRT1 activity [97, 98]. It has been postulated that health benefits of resveratrol in the gut are largely dependent on the gut microbiota.

### 3.2. Antioxidant and Anti-Inflammatory Properties of Resveratrol-Derived Microbial Metabolites

As mentioned previously, large quantities of resveratrol and its metabolites were found in the content of gastrointestinal tract in resveratrol-fed animals. The new data suggested that most unabsorbed resveratrol was transformed by gut microbiota into various bioactive metabolites, including DH-RES, 3,4'-dihydroxy-trans-stilbene, and piceid [99]. The enzymes of these bacteria, such as* Slackia equolifaciens* and* Adlercreutzia equolifaciens,* are responsible for the hydrogenation of resveratrol to yield DH-RES [100]. In addition, Jung et al. [101] showed that DH-RES was also synthesized by* E. lenta ATCC 43055 *and* B. uniformis ATCC 8492 *in vitro. For piceid, studies have demonstrated that* Bacillus cereus *could transform resveratrol into piceid. Of note, though the biological activity of resveratrol is generally attributed to the parent drug, bacterial-derived metabolites have demonstrated equal or similar activity.

In* in vitro* experiments, Lin et al. [102] indicate that 3,4'-dihydroxy-trans-stilbene has cytoprotection function against t-BHP-induced oxidative insult through activating the Keap1-Nrf2-ARE signaling pathway and the downstream antioxidant genes in HepG2 cells. This bacterial metabolite exhibits similar antioxidant effects in gut tract. Moreover, recent evidence has established that DH-RES, whose solubility is higher than that of resveratrol, can participate in the inhibition of NF-*κ*B activity in the cerulein-treated rats [103]. Piceid and resveratrol show similar activities as inhibitors of the lipid peroxidation. Piceid may be more efficacious than resveratrol due to the slow reactivity [104]. Piceid administration can alter the transfer of electrons from NADPH to oxygen and the production of ROS, increasing the availability of GSH and the activity of SOD in rotenone-induced Parkinson's disease rat models [105]. Plant callus is considered as a source of valuable secondary metabolites. Several studies have reported that rice callus suspension culture (RCSC) exhibits strong anti-inflammatory and antiproliferative activity. RCSC treatment has been shown to exhibit strong ROS modulating effects and reduce the effect of inflammation on cell death in cell lines treated with the proinflammatory cytokine cocktail. Piceid, 4-deoxyphloridzin, 5′-methoxycurcumin and lupeol, identified through HPLC and mass spectroscopy, are responsible for biological activities of RCSC [106]. Zhu et al. [107] demonstrated that DH-RES, an extract of* Dendrobium*, quenches intracellular ROS in a more efficient manner than vitamin E. In experimental acute pancreatitis rats, the levels of proinflammatory cytokines were notably reduced, and the nuclear expression of NF-*κ*B was remarkably decreased with the administration of DH-RES at 10, 20, and 50mg/kg [108]. Furthermore, Kim et al. [109] recently reported that resveratrol-FMT recipients, which are obese mice receiving FMTs from healthy resveratrol-fed mice, showed significantly lower inflammatory cytokine levels in the colon when compared with Chow-FMT recipients. These results indicated that resveratrol and/or its metabolites suppressed mucosal inflammation through the inhibition of NF-*κ*B activation. Sung et al. [110] previously demonstrated that bacterial-derived metabolites induced by resveratrol are capable of modulating energy metabolism in high-fat high-salt-fed mice receiving FMTs and increasing SCFAs production. It is suggested that the biological activity of metabolites or nonliving microorganisms in the gut is also associated with SCFAs production.

## 4. Conclusions

Gut microbiota regulates the cellular redox state in the host organism.* Lactobacillus*-mediated ROS production within gut epithelial cells in low levels maintains gut homeostasis, whereas high levels of extracellular O2− induced by* Enterococcus faecali*s cause epithelial cell DNA damage, intestinal injury, and inflammatory responses. Additionally, proinflammatory cytokines expression and the production of SCFAs, which are associated with enteric bacteria, can both modulate the proinflammatory NF-*κ*B signaling pathway.

Resveratrol can inhibit inflammatory disorders through the changes in the gut microbiota. Resveratrol and its microbial metabolites can reduce the increased levels of ROS, activate Nrf2 signaling, and improve oxidative stress. They protect epithelial barrier function and suppress the activation of NF-*κ*B and intestinal inflammation. However, questions remain regarding the bidirectional interactions between resveratrol and gut microbiota, and if these interactions can fully afford resveratrol's biological activity. The mechanism of how resveratrol and its derivatives regulate ROS production in intestinal epithelial cells has yet to be fully elucidated. Considering the health benefits of resveratrol and its metabolic characteristics, the administration of resveratrol is a novel and rational strategy for the treatment of chronic inflammatory diseases.

## Figures and Tables

**Table 1:** The effects of resveratrol administration are partly dependent on the gut microbiota.

Animal model	Treatment	Alerted bacterial taxa	Biological effects	Reference
Kunming mice	High-fat diet	Increasing *Lactobacillus* and *Bifidobacterium *abundance; decreasing *Enterococcus faecalis *abundance	Anti-obesity effects	Qiao et al. [41]

Wistar rats	High-fat sucrose diet	Decreasing the abundance of *Parabacteroides *genus	Altering the mRNA expression of tight-junction proteins and inflammation-associated genes	Etxeberria et al. [90]

Fischer F344 rats	DSS	Increasing *Lactobacillus* and *Bifidobacterium *abundance; decreasing *Enterococcus faecalis *abundance	Protecting the colonic mucosa architecture and reducing systemic inflammation markers	Larrosa et al. [84]

Wistar rats	High-fat diet	Inhibiting the growth of *Bacteroides* and *Desulfovibrionaceae sp.*; enhancing the proportion of *Blautia* and *Dorea* in the *Lachnospiraceae* family	Reducing fasting blood glucose levels and increasing the HDL-c levels	Yang et al. [91]

C57BL/6J mice	Choline or Trimethylamine	Increasing the relative abundance of *Bacteroides*, *Lactobacillus*, *Bifidobacterium*, and *Akkermansia*; decreasing the relative abundance of *Prevotella*, uncultured *Ruminococcaceae*, *Anaerotruncus*, *Alistipes*, *Helicobacter*, and uncultured *Peptococcaceae*	Anti-atherosclerosis effects	Chen et al. [93]
